# Tuning Contact Angles of Aqueous Droplets on Hydrophilic and Hydrophobic Surfaces by Surfactants

**DOI:** 10.1021/acs.jpcb.2c01599

**Published:** 2022-04-25

**Authors:** Fabio Staniscia, Horacio V. Guzman, Matej Kanduč

**Affiliations:** Department of Theoretical Physics, Jožef Stefan Institute, Ljubljana SI-1000, Slovenia

## Abstract

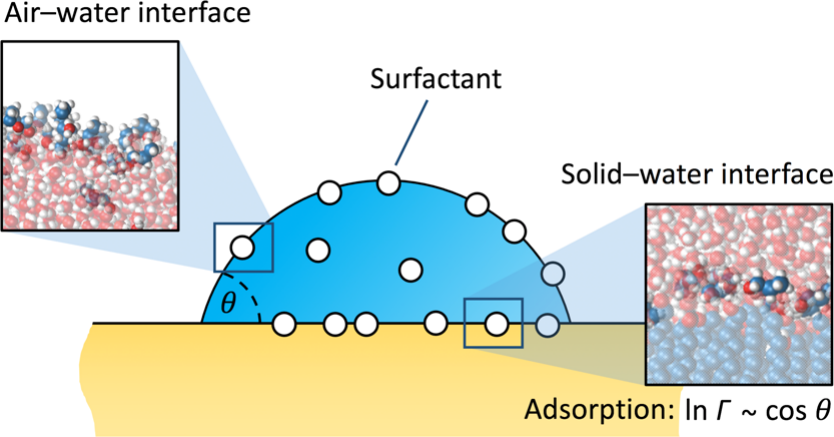

Adsorption of small amphiphilic molecules occurs in various biological and technological processes, sometimes desired while other times unwanted (e.g., contamination). Surface-active molecules preferentially bind to interfaces and affect their wetting properties. We use molecular dynamics simulations to study the adsorption of short-chained alcohols (simple surfactants) to the water–vapor interface and solid surfaces of various polarities. With a theoretical analysis, we derive an equation for the adsorption coefficient, which scales exponentially with the molecular surface area and the surface wetting coefficient and is in good agreement with the simulation results. We apply the outcomes to aqueous sessile droplets containing surfactants, where the competition of surfactant adsorptions to both interfaces alters the contact angle in a nontrivial way. The influence of surfactants is the strongest on very hydrophilic and hydrophobic surfaces, whereas droplets on moderately hydrophilic surfaces are less affected.

## 1 Introduction

Adsorption of dissolved molecules from an aqueous phase onto interfaces with air and solids is a ubiquitous phenomenon in natural and technological processes. For instance, the adsorption of organic material (e.g., microorganisms and pollen) plays a prominent role in several aspects of atmospheric and oceanic environments.^1−4^ Adsorption is essential in many applications, ranging from detergency, printing, surface catalysis, dialysis, and filtration^5^ to petrochemical processes^6^ and removal of water pollutants.^7^ Yet, adsorption is a process often challenging to predict and control. Uncontrolled adsorption contributes to surface contamination, biofouling (i.e., unwanted bacterial adhesion), loss of product to vessel surfaces, clogging of small constrictions in coronary stents^8,9^ or microfluidic devices,^10^ and deterioration of biosensors.^11^

It is known that small molecules and proteins tend to adsorb better onto hydrophobic than onto hydrophilic surfaces,^12−14^ making the latter suitable self-cleaning materials against biofouling.^9^ The water contact angle became a useful proxy for the hydrophobicity of a surface, even when addressing complex phenomena such as cellular responses to synthetic surfaces in culture media or simulated medical device service environments.^15^ However, in many complex biological scenarios, other factors become important as well.^16^ Unfortunately, the surfactant adsorption processes are challenging to study experimentally, in particular, because the adsorbing layers are typically below a few nanometers in thickness, often comprising a single molecular monolayer.^17−20^

An important effect of adsorbed molecules is that they reduce the surface tension of the interface to which they adsorb,^21,22^ which is why surfactants are often used to enhance the wetting ability of aqueous solutions^23^ and to suppress hydrophobic cavitation.^24,25^ Surface-active molecules can dramatically alter the substrate wettability, thereby leading to phenomena such as superspreading^26^ or autophobing (spontaneous retraction of a drop after initial spreading).^27,28^ Determining the relationship between the surface tension and the structures of surfactant additives at different temperatures, pressures, salinities, and pH regimes is critical for the design in many industry sectors, ranging from consumer chemicals to oil and gas extraction.^29,30^ In recent years, we have witnessed an enormous interest in surfactant-containing droplets, where the surfactant’s adsorption to the solid–water and air–water interfaces can render wetting in a nontrivial way.^31−39^

Among the vast number of additives, alcohols hold a special place, being by far the most frequently used.^40^ Short-chained alcohols are the simplest molecules that contain both hydrophobic and hydrophilic groups and are therefore excellent model systems in studies of interfaces.^41−45^ They are the most common cosurfactants added to surfactant and oil systems, for instance, in microemulsions. Alcohol adsorption is also relevant to distillation,^46^ biofuels,^47^ biomass transformation,^48^ pharmacological processes (binding to membranes and proteins),^49−51^ and aerosol science.^52,53^

**Figure 1:**
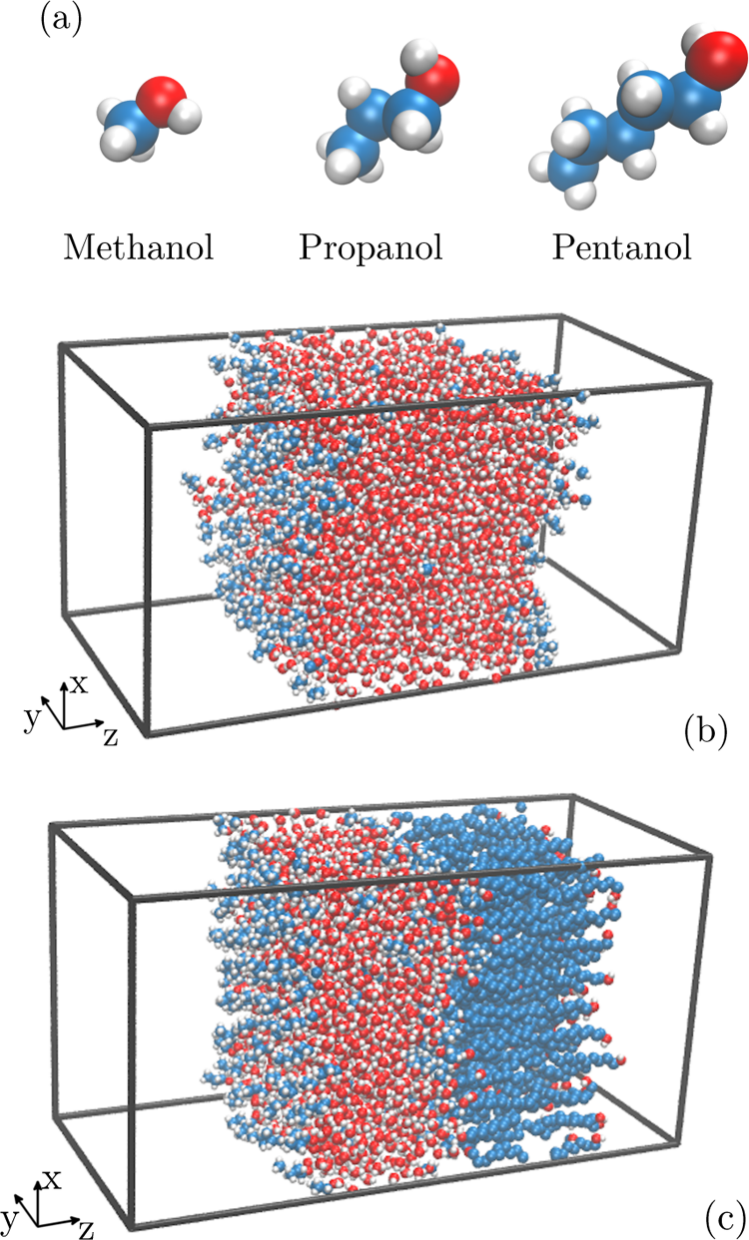
Simulation models. (a) Surfactant molecules in this work: methanol, 1-propanol, and 1-pentanol. (b) Simulation box of a water slab containing surfactant molecules, used to study the water–vapor adsorption. (c) Simulation box of a water slab in contact with the planar surface.

In this work, we employ molecular dynamics (MD) simulations to study how short-chained alcohols (i.e., methanol, 1-propanol, and 1-pentanol, shown in Figure 1a) adsorb to two kinds of interfaces: water–vapor and solid–water. For the latter, we use a self-assembled monolayer (SAM) with various degrees of polarity and water contact angles. The three linear alcohols are soluble in water,^54^ which enables studying the effect of chain length directly. Since they adsorb to both interfaces and lower their surface tension, we will refer to them also as surfactants^55^ in this work. We compute the adsorption of alcohols onto the interfaces and analyze the dependence on the chain length and the surface contact angle, θ, expressed in terms of the wetting coefficient, cos θ. We invoke a continuum-level approach to rationalize the observed relationship between the adsorption and the wetting coefficient. Furthermore, using the Gibbs adsorption-isotherm formalism, we relate the surfactant adsorption to the decrease in the surface tensions. This enables us to analyze the variation of droplet contact angles as a function of the surfactant concentration.

## 2 Methods

### 2.1 Atomistic Models

We used the simple point charge/extended model for water^56^ combined with the GROMOS force field^57^ for simulating alcohols and the solid surface. All-atom structures and topology files for alcohols were obtained from the ATB repository.^58^

To simulate the adsorption at the water–vapor interface, we set up an *NVT* (fixed number of particles, volume, and temperature) simulation with box dimensions of 5 nm × 5 nm × 10 nm with a water slab (containing various numbers of alcohol molecules) of thickness 5 nm in the middle (see Figure 1b and Section S1 in the Supporting Information for simulation details). Periodic boundary conditions were applied in all three directions. The vapor layer (of thickness 5 nm) was thick enough so that the water slab did not interfere with its periodic images along the *z* direction.

For the planar solid surface, we adopted an atomistic model introduced before,^59−61^ which mimics a SAM. The surface was composed of restrained, hexagonally packed aliphatic chains terminated by hydroxyl (OH) head groups with the area density of 4.3 nm^–2^. For the aliphatic chains, the united-atom representation was used. To generate different hydrophilicities of the surface, the original partial charges in the OH groups were scaled by the factors 0, 0.4, 0.6, 0.7, and 0.8, which resulted in the water contact angles of θ = 135, 120, 97, 76, and 45°, respectively, as determined previously by the sessile droplet method^61^ as well as thermodynamic integration.^62^ The relation between the polarity and contact angle is provided in Section S2 of the Supporting Information. A 5 nm-thick water slab with added surfactants was placed in contact with the surface. The simulation box (of height 10 nm and lateral dimensions 5.2 nm × 4.5 nm—the closest commensurable choice to the water-slab system) was replicated in all three directions via periodic boundary conditions (see Figure 1c).

### 2.2 Simulations and Data Analysis Details

The MD simulations were performed with the GROMACS 2019 simulation package.^63^ The temperature was maintained at 300 K using the velocity-rescaling thermostat^64^ with a time constant of 0.1 ps. In *NPT* (fixed number of particles, pressure, and temperature) simulations (used for the Kirkwood–Buff integrals), the pressure was controlled with the Parrinello–Rahman barostat^65,66^ of time constant 1.0 ps. Electrostatics was treated using particle-mesh-Ewald methods^67,68^ with a real-space cutoff of 0.9 nm. The Lennard-Jones potentials were cut off at 0.9 nm in order to be compatible with the previous studies that employed the same SAM model and also evaluated the contact angles.^61,62^ Simulation times spanned up to 300 ns for the water–vapor systems (three independent realizations of 100 ns, used to gather sufficient statistics for evaluations of surface tensions) and 100 ns for the surface–water systems.

When performing fits to data, we used the orthogonal distance regression algorithm,^69^ which allows us to include the uncertainty of the data in both (*x* and *y*) coordinates. This is necessary since, for some sets of data, the relative uncertainty of the *x*-coordinate is much larger than that of the *y*-coordinate.

## 3 Results and Discussions

### 3.1 Adsorption at the Water–Vapor Interface

**Figure 2:**
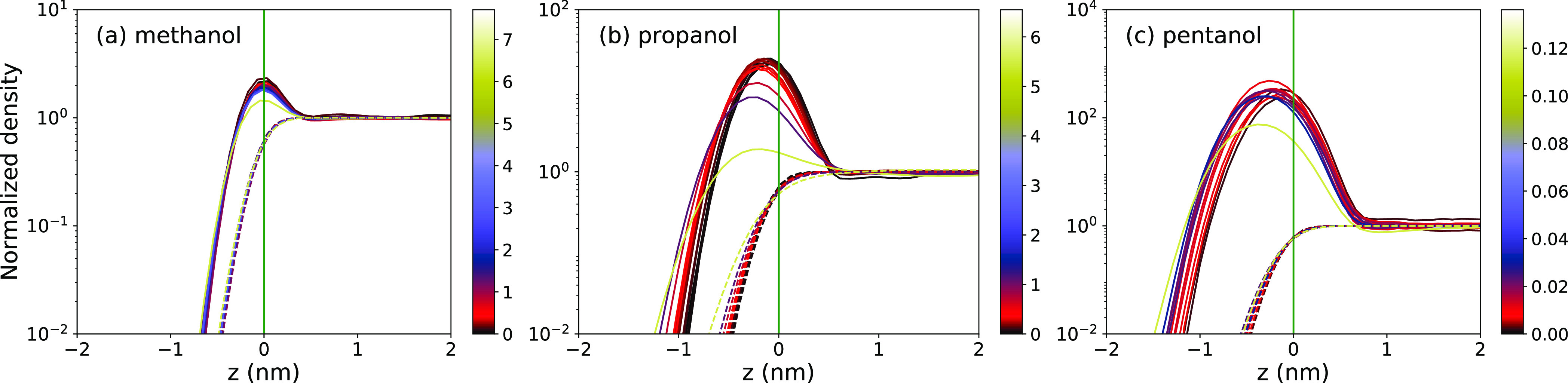
Normalized water (dashed lines) and surfactant (solid lines) density profiles (in logarithmic plot) at the water–vapor interface for different concentrations of (a) methanol, (b) propanol, and (c) pentanol. Different colors correspond to different bulk concentrations *c*_0_ of the surfactant shown by the color bar on the right in the unit of mol/l. The green vertical lines indicate the Gibbs dividing surface of the water phase.

We start by examining the adsorption behavior at the water–vapor interface (a proxy for the air–water interface), which is one of the most studied interfaces.^45,46,54^Figure 2 shows normalized density profiles of water [*c*_w_(*z*)/*c*_w0_; dashed lines] with various concentrations of surfactant [*c*(*z*)/*c*_0_; solid lines] in the proximity of the liquid–vapor interface. The pronounced density peaks of surfactants at the interface indicate preferential adsorption.

Adsorption is commonly quantified as the surface excess number density Γ of the surfactant across the effective water–vapor boundary, located at *z*_0_
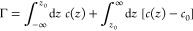
1where *c*_0_ is the bulk surfactant concentration. We define the effective position of the water–vapor interface, *z*_0_, as the Gibbs dividing surface of the water phase (i.e., the position at which the excess water adsorption vanishes). To numerically compute the above integrals, we first identify the water bulk as the region where surfactant density is roughly uniform and use it to evaluate *c*_0_ (Tables S1–S3 in the Supporting Information provide the number of simulated molecules and the bulk concentrations). We used the trapezoidal summation rule of subinterval length Δ*z* = 0.1 nm to numerically integrate density profiles *c*(*z*) from a position well inside the vapor phase to a position well inside the water phase.

**Figure 3:**
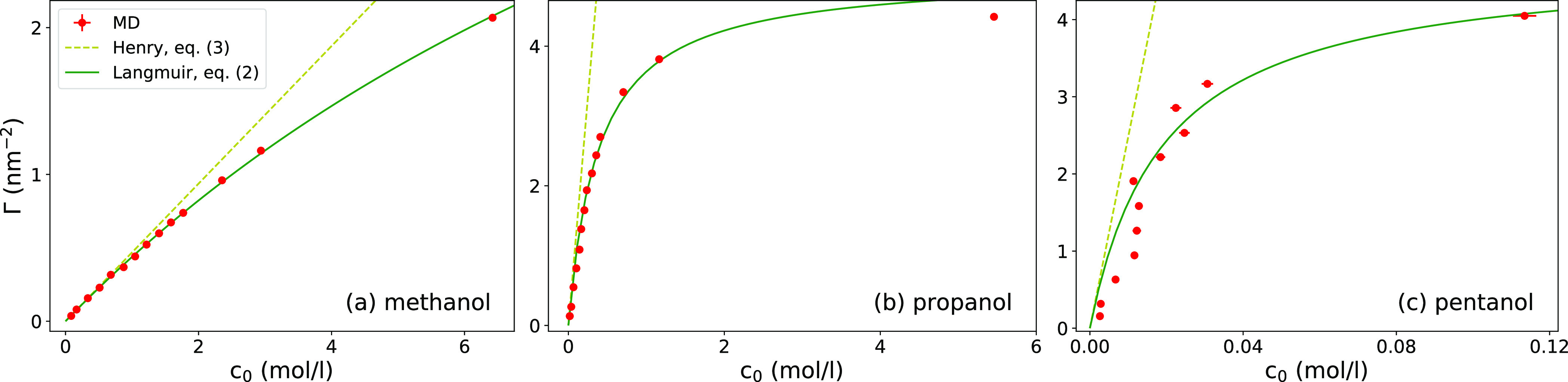
Adsorption Γ at the water–vapor interface as a function of bulk concentration of (a) methanol, (b) propanol, and (c) pentanol. The MD data (red symbols) are fitted with the Langmuir isotherm (eq 2) (green line). Yellow dashed lines correspond to Henry’s law (eq 3), for which the coefficient *K*_v_ is obtained from the Langmuir fit (*K*_v_ = *k*_c_Γ_∞_).

In Figure 3, we plot evaluated adsorptions Γ as a function of bulk concentration *c*_0_ for all three surfactants. Generally, at first, a linear trend for low concentrations starts leveling off at higher concentrations, which can be approximately described by the Langmuir adsorption isotherm^21,70−72^
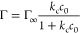
2shown in Figure 3 as green solid lines and where *k*_c_ and Γ_∞_ are fitting parameters. For low concentrations, eq 2 reduces to Henry’s law
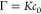
3where *K* = *k*_c_ Γ_∞_ is the adsorption coefficient. We denote it as *K*_v_ when representing the adsorption coefficient to the water–vapor interface and *K*_s_ to the solid–water interface. Henry’s law is also shown in Figure 3 as dashed lines for comparison, with the adsorption coefficient *K*_v_ as obtained from the fit of the Langmuir isotherm. The adsorption coefficient *K*_v_, as well as *k*_c_, grows rapidly with the molecular size, starting from *K*_v_ = 0.8 nm for methanol, 21 nm for propanol, and 410 nm for pentanol. Experimental values reported in the literature^21,70,73−77^ (we estimated some of the values from surface tension measurements, as described in Section S3 of the Supporting Information) are 2.1 nm (methanol), 19–32 nm (propanol), and 270–290 nm (pentanol). Thus, the MD results are capable of satisfactory reproducing experiments, given the high sensitivity on the surfactant size, as we will see later on.

In contrast, the evaluated saturation values of Γ_∞_ are comparable for the three surfactants obtained from the simulations (6.52 nm^–2^ for methanol, 5.06 nm^–2^ for propanol, and 4.80 nm^–2^ for pentanol), reflecting the fact that the adsorbed molecules occupy similar areas. Experimental data give Γ_∞_ ≃ 3.5 nm^–2^ for propanol and pentanol,^21,70^ which also compares reasonably well with our MD results. Note that the systematic accuracy of Γ_∞_ may not be very high because the fits are intentionally focused on low-concentration regimes, with few points at high concentrations.

A notable effect of surfactant adsorption at the water–vapor interface is that it reduces the surface tension, γ. The reduction can be calculated using the Gibbs adsorption equation, dγ = −Γdμ, where μ is the surfactant chemical potential. Both Γ and μ depend on surfactant concentration, *c*_0_. Whereas for Γ(*c*_0_), we assume the Langmuir isotherm (eq 2), we invoke the Kirkwood–Buff (KB) relation for the chemical potential μ(*c*_0_)^78−80^
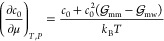
4where *k*_B_ is the Boltzmann constant, *T* is the temperature, and  and  are the molecule–molecule and molecule–water KB integrals, respectively, defined as
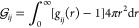
5where *g*_*ij*_(*r*) is the radial distribution function between species *i* and *j* in bulk. Evaluated *g*_*ij*_(*r*) in bulk solutions and calculated KB integrals  and  are shown in Section S4 of the Supporting Information. Since both KB integrals are nearly constant for low concentrations, we can treat them as constants. We combine eqs 2 and 4 with the Gibbs adsorption equation, and after integration, we obtain the relation between the surface tension reduction Δγ and the adsorption Γ
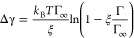
6with the correction factor
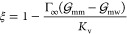
7The surface tension reduction is sometimes also expressed in terms of surface pressure π = −Δγ.

The correction factor ξ amounts to ∼0.650 for methanol, ∼0.950 for propanol, and ∼0.998 for pentanol. Let us briefly discuss the expected importance of ξ for various molecule sizes. Denoting the linear size of the molecule as *l*, Γ_∞_ roughly scales as ∼ *l*^–2^ (i.e., corresponding to the density of tightly packed monolayer of surfactants), and for nonattractive molecules (e.g., hard spheres),  (i.e., corresponding to the volume of the surfactant). The numerator of eq 7 consequently scales with the size of the molecule, . As we will see later on, the adsorption coefficient *K*_v_ in the denominator of eq 7 increases exponentially with the molecular size. Thus, the ξ correction is important only for small molecules, whereas for larger molecules, the exponentially increasing *K*_v_ makes the correction tending to unity, ξ → 1, consistent with the simulation results.

In the limit of low adsorption (i.e., Γ ≪ Γ_∞_, relevant at low concentrations), eq 6 simplifies to a linear form
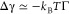
8which follows directly from Henry’s law^81^ and by assuming ideal behavior of the chemical potential. The second-order term in the above expansion is – (*k*_B_*T*ξ/2Γ_∞_)Γ^2^, from which it follows that eq 8 is expected to be valid for Γ ≪ ξ^–1^Γ_∞_ (i.e., when the second-order term is much smaller than the first term).

**Figure 4:**
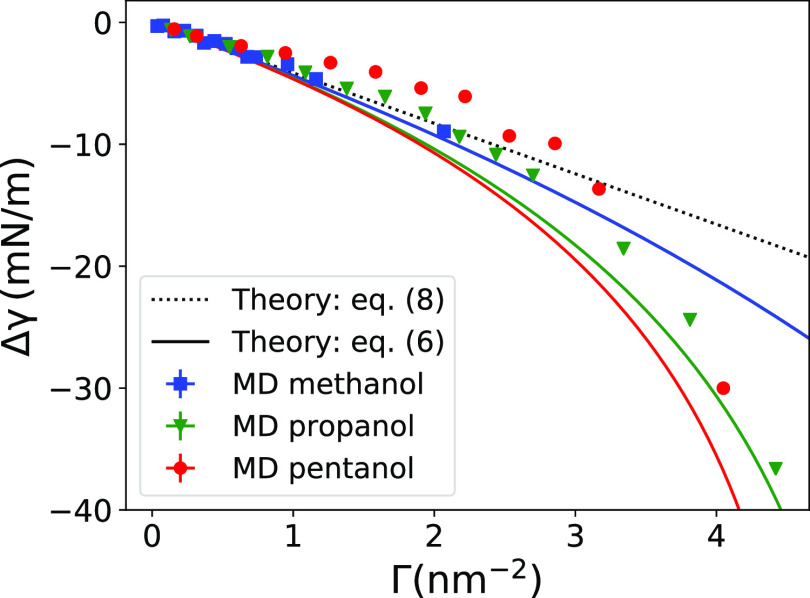
Reduction of the water–vapor surface tension versus adsorption as obtained from MD simulations (symbols) and theoretical predictions: eq 6 (solid lines) and its linear expansion eq 8 (dotted line).

Figure 4 shows the relation between the surface tension reduction Δγ and the surfactant adsorption Γ as obtained from simulations (calculated from the diagonal pressure-tensor components^82^) and theory (eqs 6 and 8). In a complementary figure in the Supporting Information (Figure S2), we compare MD simulations with experiments in terms of Δγ(*c*_0_), which shows qualitative agreement for methanol and, notably, quantitative agreement for propanol and pentanol, thereby verifying the quality of the used molecular model.

For small adsorption, the simple linear relation given by eq 8 (dotted line) matches very well the MD data in Figure 4. For higher adsorptions, the surface tension progressively sinks with adsorption, which is considerably well captured by the nonlinear relation (eq 6). However, some deviations are observed for intermediate values of Γ for propanol and, more so, pentanol. Clearly, the underlying theoretical assumptions have limitations, one of which is the use of the Langmuir isotherm, especially for fitting the pentanol data (Figure 3c).

In the Supporting Information (Section S5), we analyzed the surfactant adsorption based on the second-order virial expansion.^62,83^ The calculated values for Γ(*c*_0_) (Figure S6) and Δγ(Γ) (Figure S7) match the MD values up to the intermediate concentrations very well. The observed agreement implies that the deviations mentioned above stem from the attraction and cluster formation of surfactants at the water–vapor interface, which is not captured by the Langmuir isotherm or the theories based on them.

### 3.2 Adsorption onto Solid Surfaces

We now turn our attention to solid surfaces and investigate how changing the polarity, manifesting in different contact angles (θ ≃ 45°–135°), affects the adsorption of the three surfactants. More details are provided in the Methods section and in refs (59)–^61^.

**Figure 5:**
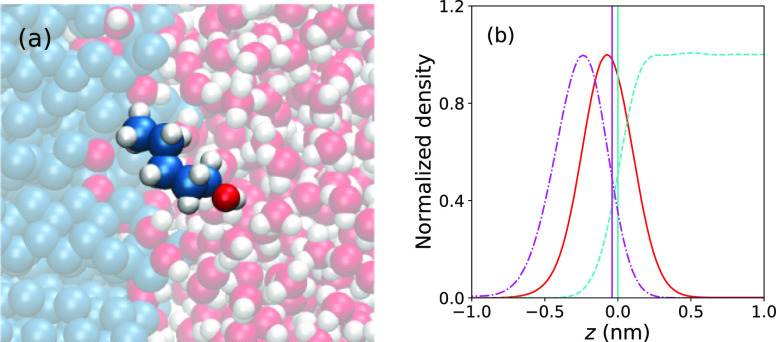
(a) Snapshot of an adsorbed pentanol molecule at the nonpolar surface (on the left in blue) with θ = 135°. (b) Corresponding rescaled density profiles of pentanol (red solid line) with bulk concentration of *c*_0_ = 0.0026 mol/l, surface OH groups (magenta dash-dotted line), and water (cyan dashed line). Effective phase boundaries are depicted by the Gibbs dividing surface for water (cyan solid line) and the position at half-height on the water side of the OH group (magenta solid line).

Figure 5a is a snapshot of a pentanol molecule adsorbed on the hydrophobic surface with θ = 135°. The molecule partially penetrates into the surface’s interior by locally deforming the neighboring surface molecules. From the density profiles of this scenario, shown in Figure 5b, we estimate that the molecule penetrates into the surface’s interior roughly by half of its size. Similar behavior is also found for the other two alcohols and other surface polarities; see Figure S8 in the Supporting Information.

**Figure 6:**
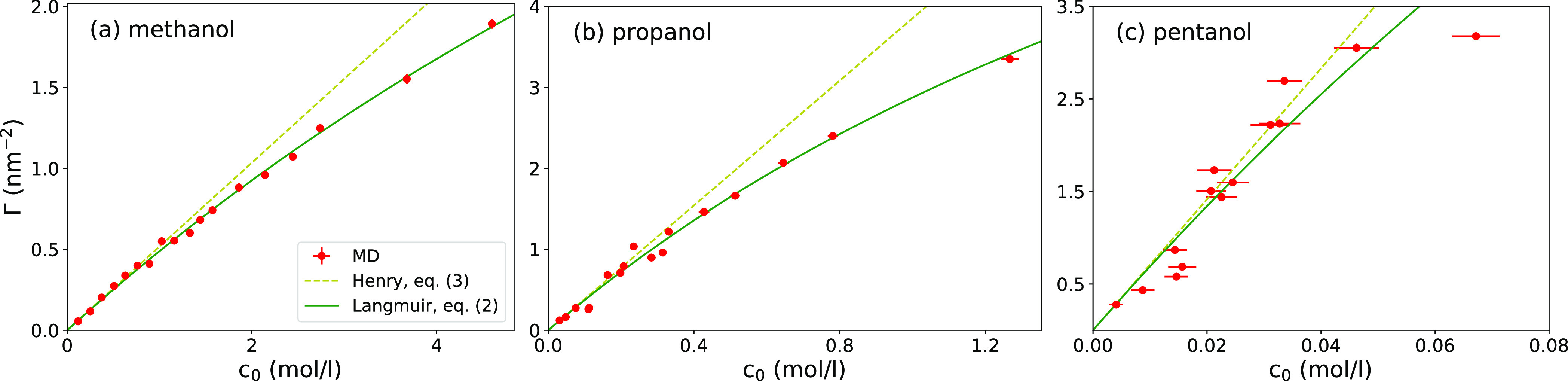
Adsorption onto the surface with a wetting contact angle of θ = 97° as a function of bulk concentration of (a) methanol, (b) propanol, and (c) pentanol. MD values are shown by red circles, whereas solid green lines show the fits of the Langmuir isotherm. Yellow dashed lines correspond to Henry’s law (eq 3), for which the coefficient *K*_v_ is taken from the Langmuir fit.

Following the same procedure as for the water–vapor adsorption, we evaluate the adsorption–concentration relations, a few representative examples of which are shown in Figure 6 for a mildly hydrophobic surface with θ = 97° (the rest can be found in Section S6 of the Supporting Information). The overall qualitative behavior is the same as at the water–vapor interface, and it can be likewise well described by the Langmuir isotherm (shown by solid lines in Figure 6). The values of Γ_∞_ are shown in Figure S12 in the Supporting Information.

**Figure 7:**
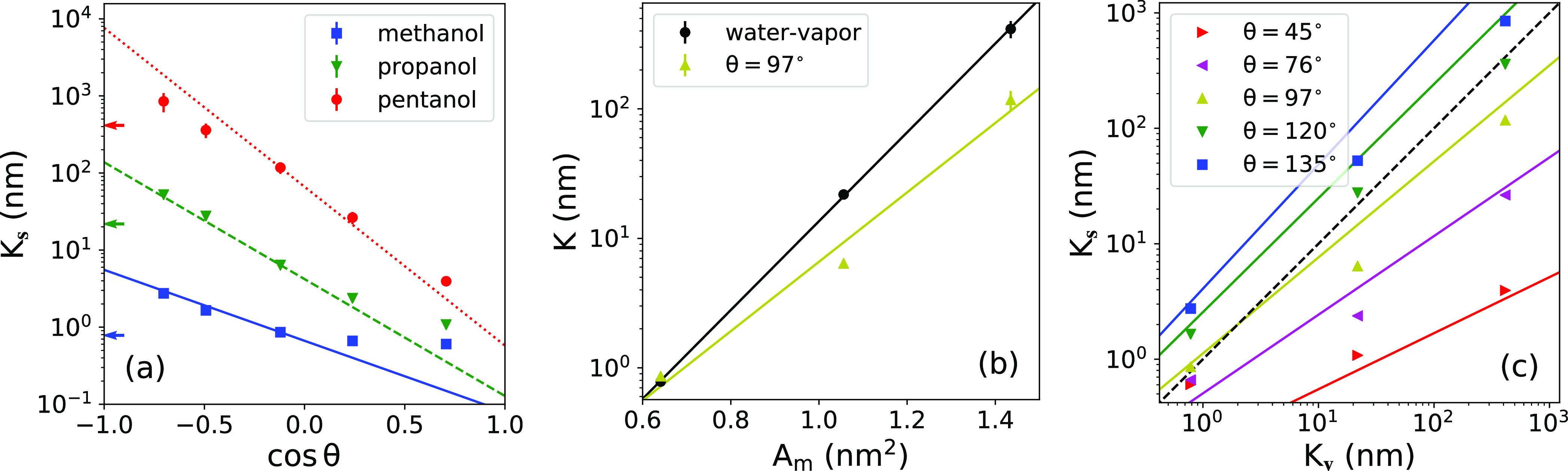
Adsorption coefficients. (a) Adsorption coefficient versus wetting coefficient of the surface for all three alcohols. The lines are predictions of eq 13, whereby the coefficient *K*_s_^(0)^ (controlling the offset) was used as a fitting parameter to the middle data points, with the wetting coefficient closest to zero (cos θ = −0.12). The arrows on the left indicate the adsorption coefficients to the water–vapor interface, *K*_v_. (b) Adsorption coefficient *K*_s_^(0)^ for the vanishing wetting coefficient versus the molecular surface area *A*_m_. A comparison with the water–vapor interface *K*_v_ is also shown. The solid lines are fitted exponential functions (eqs 14 and 15), which give γ̃_s_ ≃ 25.6 and γ̃_v_ ≃ 32.7 mN/m. (c) Correlation between adsorption coefficient to the solid surface (*K*_s_) and that to the water–vapor interface (*K*_v_). The symbols are MD results, and solid lines are predictions of eq 16. The dashed diagonal line denotes the symmetric case *K*_s_ = *K*_v_.

In Figure 7a, we plot the adsorption coefficients for the surface, *K*_s_, against the surface wetting coefficient, cos θ. The outcomes clearly show that the hydrophobic surfaces have a much higher propensity to molecular adsorption than hydrophilic surfaces, which is consistent with the overall adsorption correlation with the contact angle found in various contexts.^9,14,84,85^ Moreover, the results even suggest an approximate quantitative relation of the form ln  *K*_s_ ∼ cos θ, which we will rationalize in the following.

**Figure 8:**
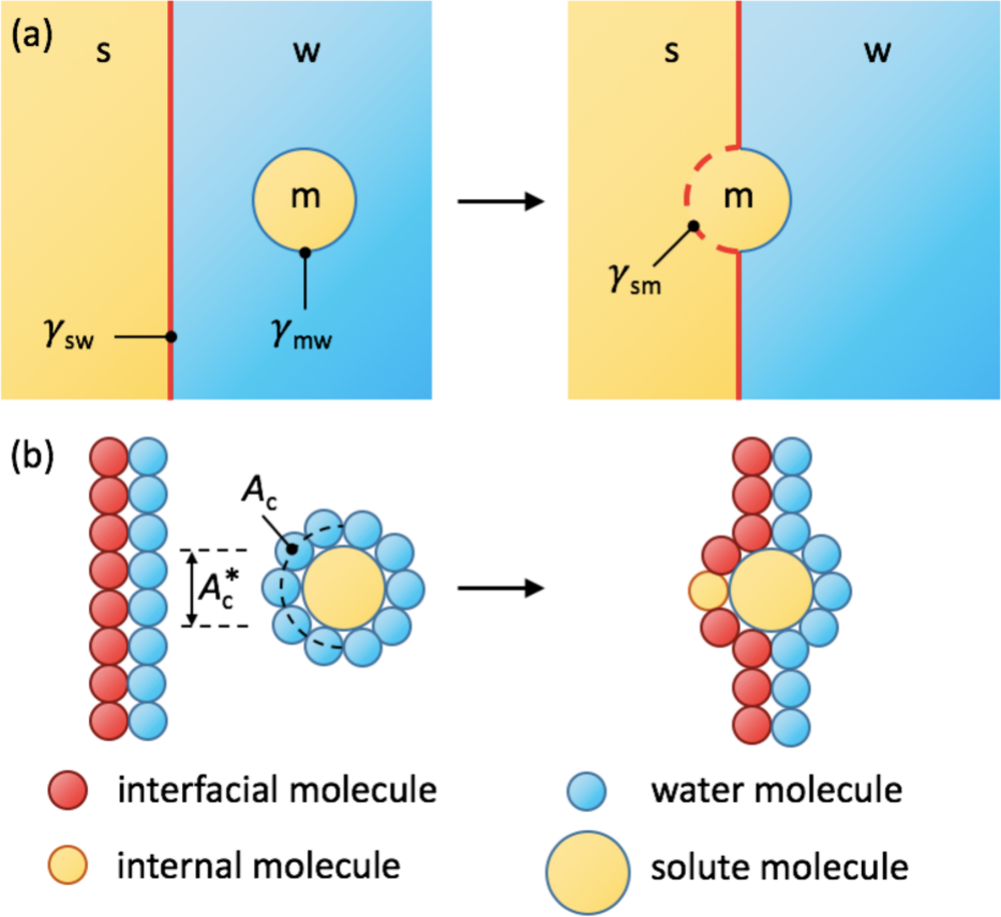
Schematic depiction of molecular adsorption to a soft surface. (a) Continuum picture: Adsorption is governed by surface tensions between the molecule (m), water (w), and the surface (s). (b) Molecular picture: The relevant areas are the bare cross-sectional surface area of the molecule (*A*_c_^*^) and the surface-accessible contact surface area (*A*_c_).

Since the adsorption increases with alkyl length, the driving mechanism should be the hydrophobic effect.^20^ In order to at least qualitatively explain the observed relation, we resort to a continuum description of adsorption, as schematically depicted in Figure 8a: A surfactant molecule (m) adsorbs from bulk water (w) to the soft surface (s) by partially penetrating inside. The free energy of this adsorption scenario is composed of two contributions. Upon adsorption, the surfactant molecule forms direct contact with the surface of area *A*_c_. In doing so, the water molecules in this area of the surfactant molecule had to be removed. The corresponding free-energy change is – *A*_c_γ_mw_, where γ_mw_ is the molecule–water surface tension. The other contribution comes from new contacts between the molecule and the surface. However, even though the overall contact area with the surface is *A*_c_, the surface area with the OH head groups is equal to the cross-sectional area of the molecule *A*_c_^*^. The surplus *A*_c_ – *A*_c_^*^ comes from the hydrocarbon groups hitherto buried inside the surface that are now exposed to the surfactant (see Figure 8b for illustration). Because the surfactant molecule is predominantly also a hydrocarbon (an alkyl chain), the surface surplus does not contribute to the excess surface free energy. The free energy contribution due to the new contacts is therefore *A*_c_^*^(γ_sm_ – γ_sw_), where γ_sm_ and γ_sw_ are solid–molecule and solid–water surface tensions, respectively. Summing up both contributions gives the adsorption free energy of the surfactant molecule in the continuum, macroscopic picture as
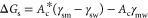
9

Figure 8b outlines the essential molecular rearrangements during the adsorption. The effective cross-sectional area *A*_c_^*^, which is the area of the removed water molecules from the surface, is best described by the cross-section of the bare molecule. If we approximate the molecule by a sphere (i.e., *A*_m_ = 4π*R*_m_^2^ and *A*_c_^*^ = π*R*_m_^2^, where *R*_m_ is its radius), the cross-sectional area is . In the other extreme limit, in which the molecule is considered as an infinitely long cylinder (i.e., *A*_m_ = 2π*R*_m_*L* and *A*_c_^*^ = 2*R*_m_*L*, where *R*_m_ is the radius and *L* is the length of the cylinder), the relation becomes *A*_c_^*^ = π^–1^*A*_m_. In cases of finite rodlike molecules (such as alcohols in our case), the ratio *A*_c_^*^/*A*_m_ lies somewhere between the two extremes of 1/4 = 0.25 and 1/π ≈ 0.32, which is a rather narrow interval. Since the continuum approach for describing molecular details is very approximate, we will assume the spherical approximation in the forthcoming analysis.

Before proceeding with eq 9, we have to be aware that applying macroscopic concepts of interfacial surface at the molecular level is in general a delicate move. Nonetheless, some problems can be, at least qualitatively, formally resolved by identifying effective molecular surface areas and curvature (i.e., Tolman) corrections to surface tensions.^86,87^ Such an analysis is, however, far beyond the scope of this study. Therefore, we will use the above continuum equation only to extract the dependence of adsorption on the contact angle. The latter is related to removal of water from the flat area of the solid, whose surface is flat (requiring no curvature corrections) and whose surface tension is macroscopically well defined.

The solid–water surface tension γ_sw_ is the only quantity in eq 9 that depends on the contact angle. The dependence is provided by the Young equation of a water droplet on the surface
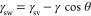
10where γ_sv_ is the solid–vapor surface tension. Equation 9 now expresses as
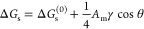
11where the reference value Δ*G*_s_^(0)^ = *A*_c_^*^(γ_sm_ – γ_sv_) – *A*_c_γ_mw_ is the adsorption free energy to the surface with a vanishing wetting coefficient, cos θ = 0 (i.e., for θ = 90°). The above equation nicely demonstrates the modulation of the adsorption free energy with the contact angle.

From a known Δ*G*_s_, the adsorption coefficient to the surface can be estimated as
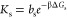
12where β = 1/*k*_B_*T* and *b*_s_ is a free parameter. Using eq 11, the dependence of the adsorption coefficient on cos θ follows as
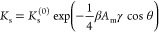
13where the reference *K*_s_^(0)^ = *b*_s_ exp(−βΔ*G*_s_^(0)^) is the adsorption coefficient for the surface with a vanishing wetting coefficient. As seen in Figure 7a, agreement between the MD data and eq 13 (with *K*_s_^(0)^ as a fitting parameter to the middle data points) is reasonably good, particularly in the hydrophobic regime (cos θ < 0). For hydrophilic cases (cos θ > 0), agreement becomes worse, especially for smaller molecules such as methanol, which feature weak adsorption. One reason for the poorer agreement is that in weakly adsorbing cases (i.e., small *K*_s_), the molecule penetrates less into the surface (see Figure S6 in the Supporting Information), and thus, *A*_c_^*^ is smaller than *A*_m_/4.

The next relevant question is, how does the reference adsorption coefficient *K*_s_^(0)^ depend on the molecular surface area. In Figure 7b, we plot the relation between *K*_s_^(0)^ and the molecular surface area *A*_m_. The result can be easily understood using eq 9, which suggests that the adsorption free energy is proportional to the molecular surface area, Δ*G*_s_^(0)^ = −γ̃_s_*A*_m_, where the proportionality coefficient γ̃_s_ can be considered as an effective molecular surface tension for adsorption.^86,87^ For the reference adsorption coefficient, we can thus write
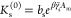
14and likewise, for the adsorption coefficient at the water–vapor interface
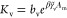
15The above two equations fit the MD data points in Figure 7b very well, with *b*_*i*_ and γ̃_*i*_ (*i* = *v*, s) used as fitting parameters.

It is insightful to look at the correlation between the adsorption coefficients to both interfaces, *K*_s_ and *K*_v_, as plotted in Figure 7c. The two coefficients are very well correlated for a given surface contact angle, implying that the better a molecule adsorbs onto the water–vapor interface, the better it adsorbs onto the solid surface. This correlation stems primarily from the linear dependence of adsorption energies on the molecular surface area. Using eqs 13–15 and eliminating *A*_m_, we come up with the following analytic relation
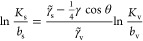
16which demonstrates that, indeed, the logarithms of the two adsorption coefficients are linearly related, with a prefactor that linearly decreases with cos θ. Using the fitted coefficients form Figure 7b, we plot the predictions of eq 16 in Figure 7c as solid lines. Even though the agreement is not perfect, the slope is nicely captured by the prefactor of eq 16, at least for the larger two alcohols.

From the correlation plot, we conclude that the adsorption to the water–vapor interface is always stronger than to the polar solid surfaces with contact angles below θ ≈ 97° — the data lie below the diagonal symmetry line. Moreover, the ratio *K*_s_/*K*_v_ becomes progressively smaller with an increasing *K*_v_ (i.e., molecular size). In contrast, the hydrophobic surfaces with contact angles above θ ≈ 120° outdo the water–vapor interface in adsorption, at least for not too large and too strongly adsorbing molecules. Same qualitative trends were experimentally observed on hydrophobic and mildly hydrophilic surfaces.^44,88^

### 3.3 Surfactant Effect on the Wetting Contact Angle

In the end, we take a look at a scenario where the adsorption to the water–vapor and a solid surface compete with each other—a sessile water droplet containing surfactants. A neat water droplet deposited on a solid surface forms the contact angle θ with the surface, given by the Young equation (eq 10). When the surfactant is introduced into the droplet, it adsorbs to both interfaces, solid–water and water–vapor, thereby reducing their surface tensions, which become dependent on the surfactant concentration (i.e., γ_sw_(c_0_) and γ(c_0_)). In principle, less-soluble surfactants can also adsorb at the solid–vapor interface,^39^ which does, however, not occur in our case (see Section S7 of the Supporting Information). Consequently, the solid–vapor surface tension, γ_sv_, remains unaffected. The Young equation of the surfactant-laden droplet then reads^27,37^
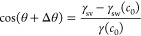
17where θ is the contact angle of the neat (surfactant-free) water droplet and Δθ is the change of the contact angle due to the surfactant. For small changes in contact angle (Δθ ≪ 1), the above equation simplifies to
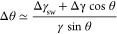
18

In the linear adsorption regime, in which Henry’s law and eq 8 apply, the expression further simplifies to
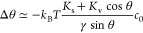
19which can also be derived from the Lucassen-Reynders equation.^89^

**Figure 9:**
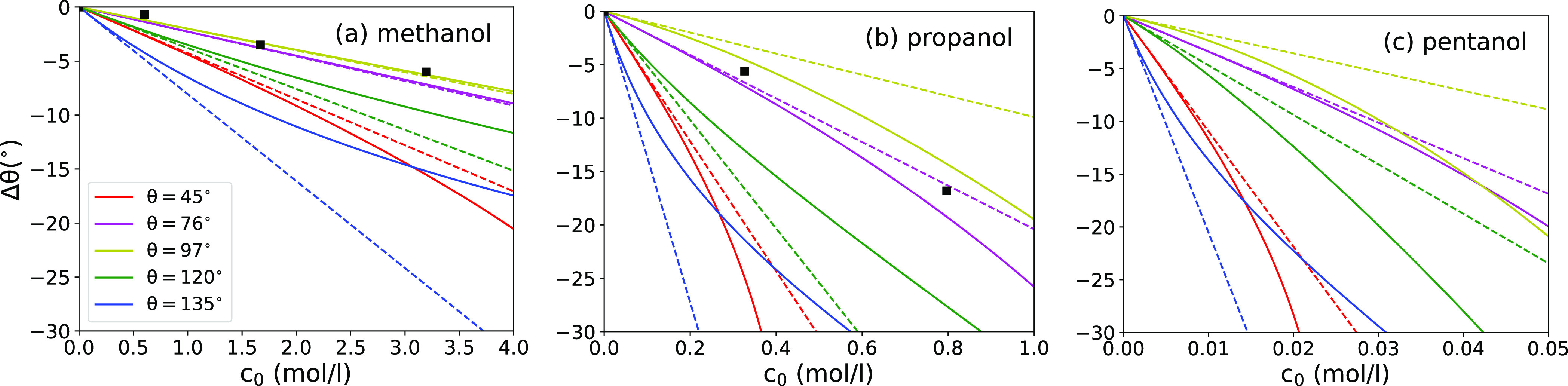
Change of the contact angle Δθ due to surfactant adsorption as a function of (a) methanol, (b) propanol, and (c) pentanol concentrations on surfaces of different contact angles. The solid lines are predictions of eq 17, and the dashed lines are low-concentration predictions given by eq 19. The black squares are experimental data for a silanized glass with θ ≃ 104° taken from ref (75), where we used the data from ref (74) to convert from molar fractions to concentrations.

In Figure 9, we show the predictions of the contact angle change for all three alcohols and for different surface hydrophilicities, based on eq 17 (solid lines) and its linearized version, eq 19 (dashed lines), along with some experimental measurements.^75^ In eq 17, we used eq 6 for calculating the surface tension reduction of both interfaces. We see that in all cases, the contact angle θ monotonically decreases with the bulk surfactant concentration in the droplet, that is, adding surfactant enhances wetting. This observation is in qualitative agreement with the Zisman plot, an empirical relation stating that cos θ linearly decreases with γ for various liquids on a given solid substrate.^32,44,75,90,91^ Experimentally measured droplet contact angles^75^ as a function of methanol and propanol concentrations on a silanized glass, which features θ ≃ 104°, show very good agreement with our results for θ = 97° (the closest value of θ we investigated). The relation of Δθ versus *c*_0_ is altogether linear at first, as predicted by eq 19, and becomes nonlinear at higher concentrations: Sublinear on hydrophobic surfaces and superlinear on hydrophilic ones.

**Figure 10:**
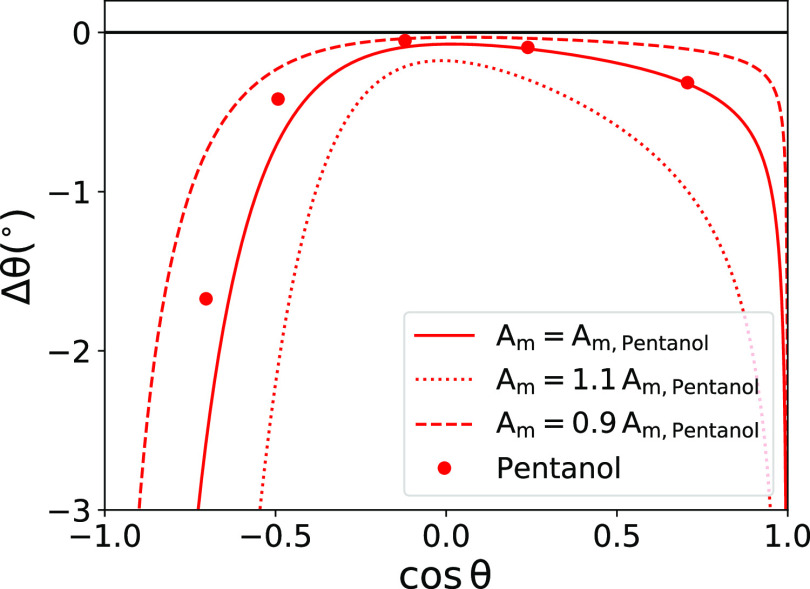
Change in the water contact angle as a function of the wetting coefficient for *c*_0_ = 0.016 mol/l of added pentanol based on eq 19. The symbols are obtained by using *K*_v_ and *K*_s_^(0)^ from the simulations of pentanol. The lines are obtained using the predictions of eqs 13, 14, and 15 for *K*_v_ and *K*_s_ and three different values for the molecular surface area *A*_m_.

Interestingly, the change in contact angle drastically and nonmonotonically depends on the surface hydrophilicity, given by cos θ, as shown in Figure 10. The nonmonotonicity results from the competition between the adsorptions onto the water–vapor and solid–water interfaces of the droplet, which is encoded in the numerator of eq 19, reading *K*_s_(θ) + *K*_v_ cos θ. On considerably hydrophilic surfaces (small θ), the adsorption of surfactants onto the surface is negligible (i.e., *K*_s_ ≪ *K*_v_), and thus, the surfactant effect is dominated by the adsorption onto the water–vapor interface, dictated by the term *K*_v_ cos θ in eq 19. In this regime, the change in the contact angle scales as Δθ ∝ – cot θ. The effect of surfactant becomes extremely large for small contact angles, and it even diverges as the surface approaches the complete wetting regime (θ → 0°). In other terms, already low concentrations of surfactant in a low-contact angle droplet can easily push the droplet into the complete wetting regime. This observation also suggests that measurements of small contact angles are particularly challenging because of potential contamination of aqueous systems with surface-active molecules.^21,92,93^

With increasing hydrophobicity (increasing θ), the surface adsorption coefficient *K*_s_ rapidly increases (see Figure 7a and eq 13) and eventually exceeds *K*_v_. Thus, |Δθ| starts dramatically rising with the surface hydrophobicity. Our analysis also shows that surfaces with contact angles around θ = 90° are the least sensitive to wetting alterations due to surfactants as compared to very hydrophilic or hydrophobic surfaces.

Remarkably, the net effect of adding simple alcohols to water is always to decrease the contact angle of the droplet (Δθ < 0), even though this is not strictly imposed by eq 19. Moreover, most experimental studies show that surfactants decrease the contact angle of aqueous solutions on hydrophobic surfaces.^22,44,88^ Theoretically, the effect could be positive (Δθ > 0) for hydrophobic surfaces (for which cos θ < 0) if the adsorption onto the surface remains small, such that *K*_s_ > – *K*_v_ cos θ, which is, however, not the case in our systems.

## 4 Conclusions

In this MD simulation study, we showed that the adsorption of simple surfactants (short-chained alcohols) to water–vapor and solid–water interfaces approximately follows the Langmuir adsorption isotherm. Smaller deviations at intermediate concentrations are found in the case of pentanol, which we attribute to attractive interactions between the surfactants. The adsorption coefficient of surfactants to solid surfaces scales roughly exponentially with the surfactant’s cross-section and the surface wetting coefficient (eq 13). The observed dependence arises from the free energy of removing the water molecules from the surface area onto which the surfactant adsorbs after that, as corroborated by the continuum approach. This finding is in accordance with widely reported observations that hydrophobic surfaces are much more prone to adsorption than hydrophilic surfaces. We applied our quantitative findings to water droplets and found that adding short-chained surfactants in all cases reduces the contact angle and enhances wetting. Our predictions also agree well with experimental studies. Such wetting enhancement depends drastically and nonmonotonically on the wetting coefficient. The highest sensitivity of the contact angle on the surfactant concentration is found on very hydrophilic and very hydrophobic surfaces, which stems from two distinct effects. On hydrophilic surfaces, the effect is due to the adsorption onto the air–water interface, whereas on hydrophobic surfaces, it is due to the adsorption onto the solid–water interface. In contrast, mildly polar surfaces, with contact angles around 90°, are the least sensitive to wetting alterations. Our findings can be applied to other well-soluble, short-chained surfactants for promoting liquid spreading, treating, or preventing bubble formation and for self-cleaning processes by aqueous drops.^31,94^ Finally, making surface-active molecules charged brings about numerous electrochemical phenomena, manifested, for instance, in zeta potential, nanobubble stability, and Jones–Ray effect,^95^ which delineate interesting research routes for future studies.

## Supporting Information Available

Composition of simulated systems; relation between the polarity rescaling factor and the water contact angle of the SAM; dependence of surface tension on the concentration; Kirkwood–Buff integrals; second-order virial expansion; adsorption to solid surfaces; and estimating surfactant adsorption at the solid–vapor interface (PDF)
