# Supplementary material for: Tuning Contact Angles of Aqueous Droplets on Hydrophilic and Hydrophobic Surfaces by Surfactants
Source: J Phys Chem B. 2022 Apr 25;126(17):3374–84. doi: 10.1021/acs.jpcb.2c01599 (PMC9082615; doi:10.1021/acs.jpcb.2c01599)
Supplement: Supplementary file 1 — jp2c01599_si_001.pdf [file jp2c01599_si_001.pdf]

Supporting Information: Tuning Contact Angles of Aqueous Droplets on Hydrophilic and Hydrophobic Surfaces by Surfactants

Fabio Staniscia, Horacio V. Guzman, and Matej Kanduč*

Department of Theoretical Physics, Jožef Stefan Institute, SI-1000 Ljubljana, Slovenia

E-mail: matej.kanduc@ijs.si

S1. COMPOSITION OF SIMULATED SYSTEMS

Tables S1–S6 provide the compositions of the simulated systems in terms of the number of used alcohol and water molecules as well as the resulting alcohol concentrations c_0 in the bulk region.

Table S1: Number of methanol molecules, water molecules, and bulk concentrations for simulations of the water–vapor interface.

N methanol	N water	c_0 (mol/l)
8	4033	0.085 ± 0.001
16	4015	0.166 ± 0.002
32	3968	0.335 ± 0.003
48	3926	0.512 ± 0.003
64	3883	0.681 ± 0.003
80	3833	0.874 ± 0.004
96	3808	1.047 ± 0.005
112	3765	1.218 ± 0.005
128	3726	1.406 ± 0.005
144	3686	1.585 ± 0.006
160	3662	1.769 ± 0.006
208	3517	2.354 ± 0.006
256	3407	2.938 ± 0.007
512	2833	6.422 ± 0.008

S2. RELATION BETWEEN THE POLARITY RESCALING FACTOR AND THE WATER CONTACT ANGLE OF THE SAM

We tuned the hydrophobicity of the SAM by rescaling the original partial charges in the OH groups. The relation between the rescaling factor, the contact angle of water droplets, and its cosine is shown in Figure S1.

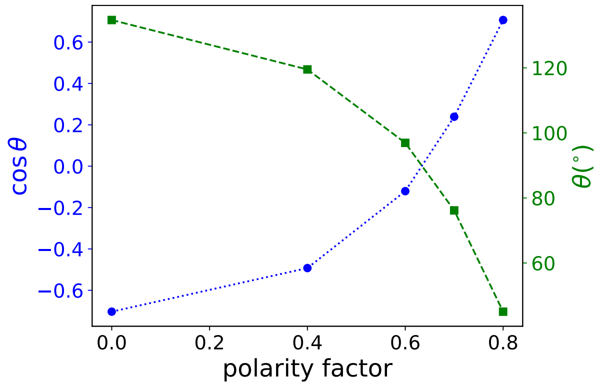

Figure S1: Water contact angle θ (green squares, right scale) and its cosine (blue circles, left scale) *versus* polarity rescaling factor of the OH groups. Data taken from Ref. 1.

S3. DEPENDENCE OF SURFACE TENSION ON CONCENTRATION

We express eq 6 in the main text in terms of bulk surfactant concentration, obtaining

$$\Delta\gamma = -\frac{k_\mathrm{B}\, T\, \Gamma_\infty}{\xi}\, \ln\left(1 - \frac{\xi\, k_c\, c_0}{1 + k_c\, c_0}\right) \tag{S1}$$

which in the linear form becomes

$$\Delta\gamma = -k_\mathrm{B}\, T\, K_\mathrm{v}\, c_0 \tag{S2}$$

Both equations are used in Figure S2, showing $\Delta\gamma$ as a function of c_0. The theoretical and MD values are compared with experimental data. The comparison shows decent agreement of the simulation data with experimental results, especially for propanol and pentanol. Linear fits (eq S2) to the experimental data give $K_\mathrm{v} = 2.1$ nm for methanol, $K_\mathrm{v} = 19$ nm for propanol, and $K_\mathrm{v} = 290$ nm for pentanol.

Table S2: Number of propanol molecules, water molecules, and bulk concentrations for simulations of the water–vapor interface.

N propanol	N water	c_0 (mol/l)
8	4018	0.018 ± 0.001
16	3984	0.037 ± 0.001
32	3911	0.066 ± 0.002
48	3841	0.103 ± 0.002
64	3759	0.143 ± 0.003
80	3703	0.165 ± 0.003
96	3616	0.206 ± 0.003
112	3542	0.237 ± 0.003
128	3466	0.302 ± 0.004
144	3395	0.357 ± 0.004
160	3328	0.410 ± 0.004
208	3113	0.705 ± 0.004
256	2921	1.164 ± 0.005
512	1904	5.459 ± 0.013

Table S3: Number of pentanol molecules, water molecules, and bulk concentrations for simulations of the water–vapor interface.

N pentanol	N water	c_0 (mol/l)
8	4000	0.0026 ± 0.0005
16	3944	0.0028 ± 0.0005
32	3844	0.0067 ± 0.0007
48	3741	0.012 ± 0.001
64	3624	0.012 ± 0.001
80	3523	0.013 ± 0.001
96	3417	0.011 ± 0.001
112	3324	0.018 ± 0.001
128	3230	0.025 ± 0.001
144	3111	0.022 ± 0.001
160	3039	0.031 ± 0.001
208	2736	0.113 ± 0.003

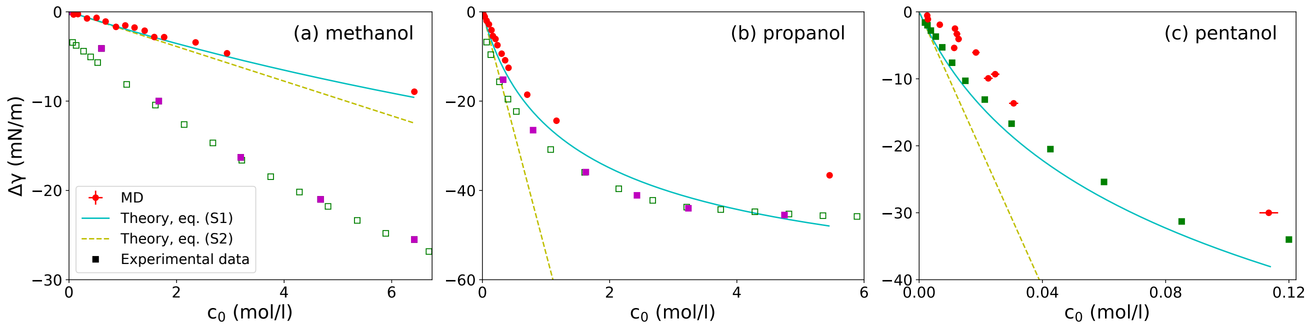

Figure S2: Reduction of the water–vapor surface tension *versus* bulk alcohol concentration as obtained from MD simulations (red circles), the theoretical predictions of eq S1 (solid lines) and eq S2 (dashed lines), and experiments (squares). The experimental data for methanol and propanol are taken from Refs. 2 (empty squares) and 3 (filled magenta squares), while for pentanol (filled green squares) they are taken from Refs. 4,5.

Table S4: Number of methanol molecules, water molecules, and bulk concentrations for simulations of the system in contact with the surface. The second column provides the number of water molecules for the simulations with $\theta = 135°$. In the simulations with other values of θ, the number of water molecules is very similar.

		$\theta = 135°$	$\theta = 120°$	$\theta = 97°$	$\theta = 76°$	$\theta = 45°$
N methanol	N water	c_0 (mol/l)	c_0 (mol/l)	c_0 (mol/l)	c_0 (mol/l)	c_0 (mol/l)
8	2320	0.086 ± 0.003	0.104 ± 0.004	0.118 ± 0.003	0.131 ± 0.003	0.123 ± 0.003
16	2296	0.169 ± 0.005	0.210 ± 0.005	0.250 ± 0.005	0.261 ± 0.004	0.258 ± 0.004
24	2272	0.282 ± 0.005	0.323 ± 0.006	0.374 ± 0.006	0.398 ± 0.006	0.399 ± 0.006
32	2258	0.366 ± 0.007	0.448 ± 0.006	0.507 ± 0.007	0.539 ± 0.006	0.545 ± 0.006
40	2230	0.464 ± 0.006	0.551 ± 0.008	0.628 ± 0.007	0.652 ± 0.006	0.673 ± 0.007
48	2218	0.573 ± 0.008	0.657 ± 0.008	0.760 ± 0.008	0.804 ± 0.008	0.797 ± 0.008
56	2201	0.669 ± 0.008	0.788 ± 0.009	0.890 ± 0.008	0.923 ± 0.009	0.937 ± 0.008
64	2174	0.801 ± 0.009	0.914 ± 0.009	1.024 ± 0.008	1.056 ± 0.008	1.083 ± 0.009
72	2164	0.913 ± 0.008	1.039 ± 0.011	1.158 ± 0.008	1.217 ± 0.010	1.239 ± 0.010
80	2146	1.020 ± 0.010	1.147 ± 0.009	1.328 ± 0.010	1.402 ± 0.008	1.383 ± 0.010
88	2126	1.141 ± 0.010	1.263 ± 0.011	1.441 ± 0.011	1.514 ± 0.010	1.523 ± 0.010
96	2094	1.258 ± 0.010	1.410 ± 0.011	1.574 ± 0.010	1.657 ± 0.011	1.680 ± 0.011
112	2059	1.485 ± 0.011	1.669 ± 0.012	1.860 ± 0.012	1.920 ± 0.011	1.968 ± 0.012
128	2031	1.761 ± 0.014	1.928 ± 0.012	2.142 ± 0.012	2.248 ± 0.012	2.272 ± 0.013
144	2016	2.022 ± 0.012	2.188 ± 0.013	2.444 ± 0.015	2.544 ± 0.012	2.620 ± 0.013
160	1954	2.340 ± 0.014	2.489 ± 0.013	2.739 ± 0.015	2.863 ± 0.014	2.873 ± 0.015
208	1905	3.179 ± 0.012	3.369 ± 0.015	3.677 ± 0.017	3.800 ± 0.016	3.854 ± 0.015
256	1774	4.170 ± 0.016	4.313 ± 0.016	4.600 ± 0.014	4.871 ± 0.017	4.845 ± 0.017

Table S5: Same as Table S4 but for propanol.

		$\theta = 135°$	$\theta = 120°$	$\theta = 97°$	$\theta = 76°$	$\theta = 45°$
N propanol	N water	c_0 (mol/l)	c_0 (mol/l)	c_0 (mol/l)	c_0 (mol/l)	c_0 (mol/l)
8	2301	0.009 ± 0.002	0.015 ± 0.002	0.031 ± 0.003	0.046 ± 0.005	0.031 ± 0.004
16	2260	0.016 ± 0.002	0.027 ± 0.003	0.048 ± 0.005	0.064 ± 0.004	0.074 ± 0.005
24	2234	0.034 ± 0.004	0.044 ± 0.004	0.075 ± 0.005	0.089 ± 0.006	0.084 ± 0.006
32	2188	0.055 ± 0.004	0.056 ± 0.005	0.110 ± 0.007	0.116 ± 0.006	0.126 ± 0.007
40	2148	0.048 ± 0.005	0.074 ± 0.005	0.113 ± 0.006	0.157 ± 0.008	0.187 ± 0.009
48	2118	0.064 ± 0.005	0.089 ± 0.006	0.163 ± 0.009	0.196 ± 0.007	0.219 ± 0.010
56	2071	0.093 ± 0.007	0.092 ± 0.007	0.198 ± 0.009	0.235 ± 0.008	0.233 ± 0.009
64	2041	0.093 ± 0.006	0.133 ± 0.007	0.207 ± 0.009	0.273 ± 0.010	0.274 ± 0.010
72	2024	0.122 ± 0.007	0.153 ± 0.009	0.235 ± 0.010	0.300 ± 0.010	0.325 ± 0.009
80	1977	0.127 ± 0.006	0.179 ± 0.009	0.283 ± 0.012	0.340 ± 0.011	0.389 ± 0.011
88	1949	0.134 ± 0.008	0.180 ± 0.009	0.314 ± 0.011	0.413 ± 0.012	0.457 ± 0.013
96	1913	0.162 ± 0.008	0.226 ± 0.010	0.331 ± 0.010	0.471 ± 0.011	0.516 ± 0.011
112	1873	0.206 ± 0.010	0.268 ± 0.010	0.428 ± 0.015	0.566 ± 0.015	0.666 ± 0.016
128	1782	0.288 ± 0.011	0.314 ± 0.010	0.513 ± 0.014	0.711 ± 0.017	0.866 ± 0.017
144	1720	0.381 ± 0.011	0.423 ± 0.011	0.645 ± 0.016	0.893 ± 0.018	1.134 ± 0.017
160	1649	0.493 ± 0.012	0.519 ± 0.013	0.781 ± 0.014	1.047 ± 0.018	1.386 ± 0.021
208	1474	0.968 ± 0.016	1.035 ± 0.018	1.267 ± 0.024	1.693 ± 0.028	2.160 ± 0.031

Table S6: Same as Table S4 but for pentanol.

		$\theta = 135°$	$\theta = 120°$	$\theta = 97°$	$\theta = 76°$	$\theta = 45°$
N pentanol	N water	c_0 (mol/l)	c_0 (mol/l)	c_0 (mol/l)	c_0 (mol/l)	c_0 (mol/l)
8	2289	0.0016 ± 0.0008	0.0019 ± 0.0008	0.0041 ± 0.0012	0.0044 ± 0.0013	0.0031 ± 0.0011
16	2231	0.0015 ± 0.0010	0.0034 ± 0.0012	0.0087 ± 0.0020	0.0199 ± 0.0029	0.0079 ± 0.0017
24	2175	0.0017 ± 0.0006	0.0055 ± 0.0015	0.0146 ± 0.0021	0.0330 ± 0.0037	0.0233 ± 0.0040
32	2144	0.0064 ± 0.0017	0.0056 ± 0.0019	0.0157 ± 0.0025	0.0226 ± 0.0037	0.0276 ± 0.0038
40	2098	0.0022 ± 0.0008	0.0067 ± 0.0019	0.0144 ± 0.0022	0.0368 ± 0.0042	0.0215 ± 0.0041
48	2020	0.0044 ± 0.0011	0.0103 ± 0.0022	0.0207 ± 0.0026	0.0266 ± 0.0031	0.0433 ± 0.0066
56	1972	0.0054 ± 0.0013	0.0131 ± 0.0026	0.0226 ± 0.0027	0.0548 ± 0.0043	0.0554 ± 0.0066
64	1919	0.0080 ± 0.0018	0.0095 ± 0.0021	0.0245 ± 0.0028	0.0538 ± 0.0042	0.1140 ± 0.0172
72	1889	0.0070 ± 0.0016	0.0103 ± 0.0022	0.0213 ± 0.0030	0.0602 ± 0.0053	0.2490 ± 0.0339
80	1834	0.0070 ± 0.0016	0.0113 ± 0.0022	0.0311 ± 0.0035	0.0781 ± 0.0077	0.2380 ± 0.0362
88	1775	0.0069 ± 0.0016	0.0153 ± 0.0021	0.0327 ± 0.0036	0.0961 ± 0.0087	0.1699 ± 0.0251
96	1737	0.0182 ± 0.0024	0.0162 ± 0.0024	0.0336 ± 0.0031	0.1409 ± 0.0120	0.3463 ± 0.0433
112	1615	0.0232 ± 0.0036	0.0161 ± 0.0022	0.0462 ± 0.0039	0.1956 ± 0.0211	0.5400 ± 0.0570
128	1540	0.0402 ± 0.0037	0.0355 ± 0.0035	0.0672 ± 0.0042	0.2547 ± 0.0254	1.4361 ± 0.0902

S4. KIRKWOOD–BUFF INTEGRALS

To calculate the bulk properties necessary for the Kirkwood–Buff (KB) theory used in Section 3.1, we simulated three independent realizations of a homogeneous system in a cubic simulation box of edge 5 nm in the NPT ensemble for 100 ns for each concentration of surfactant. From the simulations, we extracted the radial distribution functions $g_{\mathrm{mw}}(r)$ and $g_{\mathrm{mm}}(r)$, shown in Figure S3, and use them in eq 5 to calculate the KB integrals, $\mathcal{G}_{\mathrm{mw}}$ and $\mathcal{G}_{\mathrm{mm}}$, shown in Figure S4 as a function of surfactant concentration. Both $\mathcal{G}_{\mathrm{mw}}$ and $\mathcal{G}_{\mathrm{mm}}$ are independent of concentration at low concentrations (within the numerical uncertainty), which justifies our approximation in the main text.

S5. SECOND-ORDER VIRIAL EXPANSION

To rationalize the theoretical deviations from simulations observed in Figures 3 and 4, we quantified the interaction between surfactant molecules at the water–vapor interface and estimate its effect on adsorption isotherms. To that end, we simulated two surfactant molecules at a water–vapor interface for 70 ns. The two molecules were weakly restrained to the water–vapor interface by harmonic potentials of strength 3.011 mN/m in z-direction in order to prevent them from leaving the interface and simplify the analysis. From the simulations, we evaluated the two-dimensional radial distribution function between both surfactant molecules $g_{\mathrm{mm}}^{(2\mathrm{D})}(r)$ in the plane of the interface (shown in Figure S5), and used it to calculate the two-dimensional second virial coefficient

$$B_2^{(2\mathrm{D})} = -\pi \int_0^\infty \left[g_{\mathrm{mm}}^{(2\mathrm{D})}(r) - 1\right] r\,\mathrm{d}r \qquad \text{(S3)}$$

The resulting numerical values of $B_2^{(2\mathrm{D})}$ are 0.026 ± 0.008 nm^2 for methanol, -0.16 ± 0.11 nm^2 for propanol, and -0.64 ± 0.11 nm^2 for pentanol. The negative signs in the latter two indicate the effective surfactant–surfactant attraction at the water–vapor interface.

In the second-order virial expansion (valid for $B_2^{(2\mathrm{D})}\Gamma \ll 1$), the excess adsorption is given by[6,7]

$$\Gamma = K_{\mathrm{v}} c_0 - 2B_2^{(2\mathrm{D})} K_{\mathrm{v}}^2 c_0^2 \qquad \text{(S4)}$$

Figure S6 shows MD data for adsorption (similar to Figure 3 in the main text, but the fits of Henry's law involve only the first five data points). The resulting K_{v} values, which slightly differ from those calculated from the Langmuir fit, are used in eq S4, shown as dashed lines. Because of $B_2^{(2\mathrm{D})} < 0$ for propanol and pentanol, the predicted adsorption curves bend upwards. This can qualitatively explain the distinct trend for pentanol at intermediate concentrations, which features upward concavity prior to saturation at higher concentrations.

Equation S4 can now be used with the formalism of the Gibbs adsorption isotherm to calculate the surface tension reduction expressed by Γ,

$$\Delta\gamma = -k_{\mathrm{B}}T\,\Gamma - k_{\mathrm{B}}T B_2^{(2\mathrm{D})}\Gamma^2 \qquad \text{(S5)}$$

which resembles the pressure of 2D gas in the classical virial expansion. For propanol and pentanol, the correction is positive, meaning smaller reduction in γ. Figure S7 shows the surface tension reduction versus Γ (similar to Figure 4 in the main text), where the predictions of eq S5 are shown by dash-dotted lines. The predictions capture well the intermediate concentrations. This implies that the observed deviations from eq 6 (based on fitting the Langmuir isotherm) can be ascribed to attraction and cluster formation of surfactants at the interface.

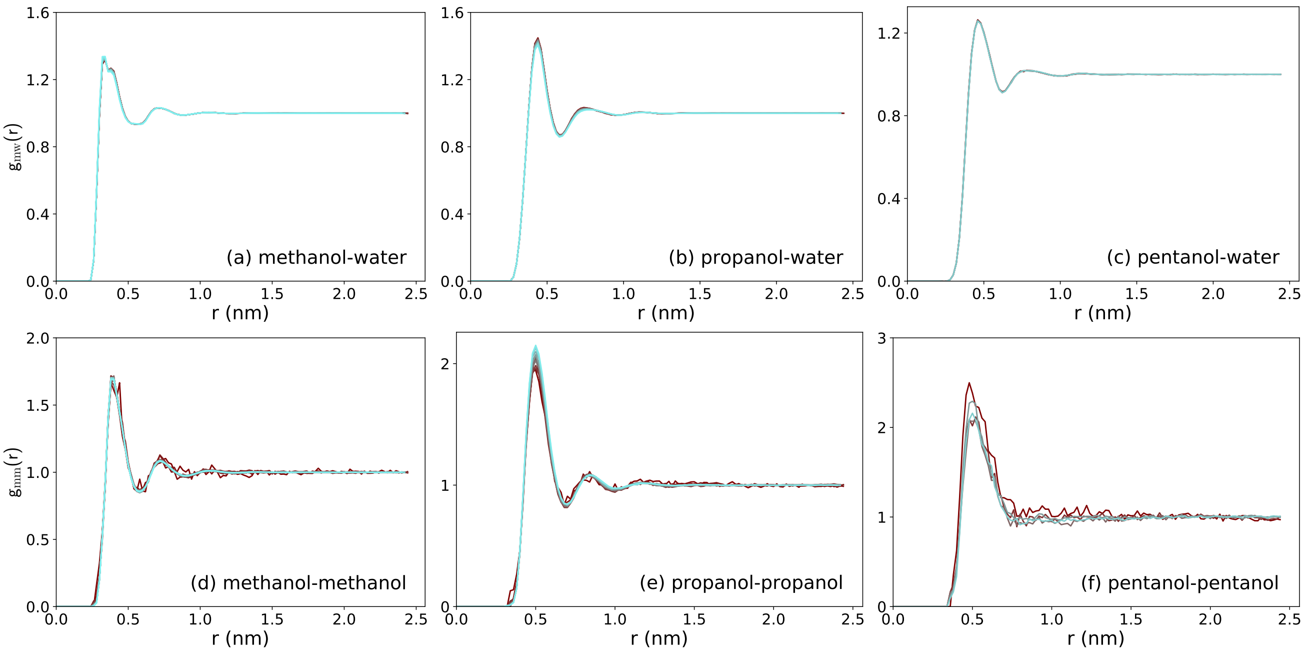

Figure S3: Radial distribution functions between water and surfactant molecules (top row) and between surfactant molecules themselves (bottom row) in a bulk solution. Different surfactant densities are shown in different colors, ranging from dark maroon (lowest) to cyan (highest).

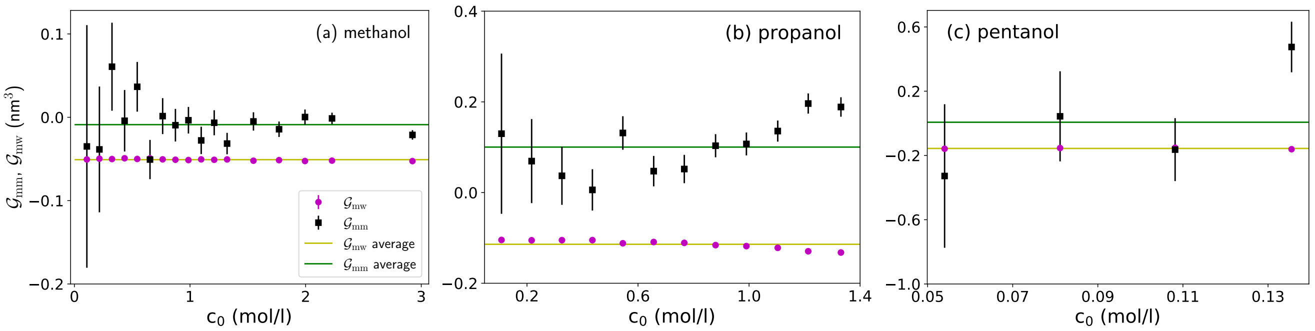

Figure S4: Kirkwood–Buff integrals, $\mathcal{G}_{mw}$ and $\mathcal{G}_{mm}$, calculated from eq 5 in the main text from the data in Figure S3 as a function of surfactant concentration. The horizontal solid lines indicate averaged values for each data set.

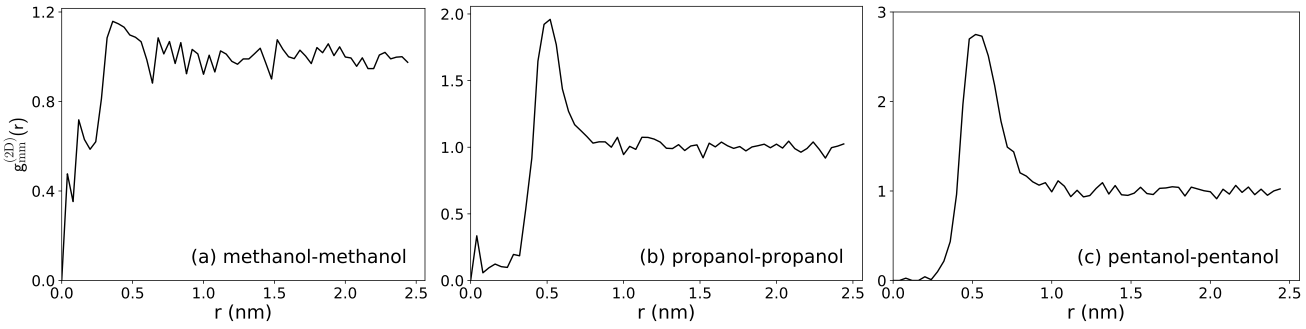

Figure S5: 2D radial distribution functions between two surfactant molecules at the water–vapor interface.

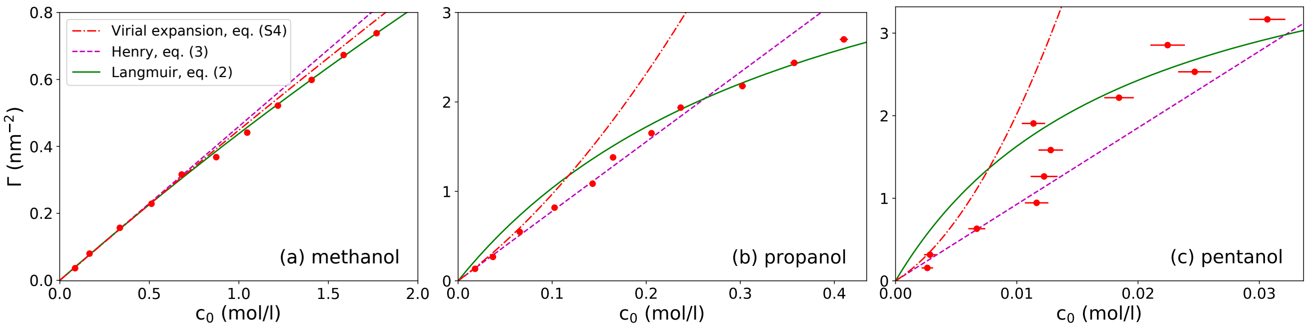

Figure S6: Adsorption Γ at the water–vapor interface as a function of the bulk concentration of (a) methanol, (b) propanol, and (c) pentanol (same MD data as in Figure 3 in the main text). Here, Henry's law is fitted to the first 5 data points (dashed lines), and the obtained K_v (0.76 nm for methanol, 12.9 nm for propanol, and 154 nm for pentanol) is used to plot the second-order virial correction given by eq S4 (dash-dotted lines). For comparison, fits of the Langmuir isotherm (eq 2) are shown as green lines.

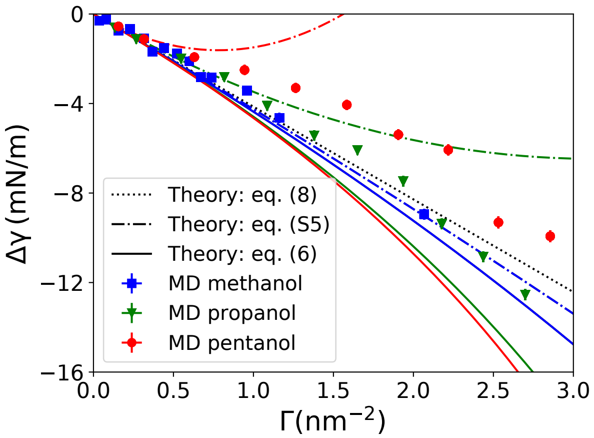

Figure S7: Reduction of the water–vapor surface tension *versus* adsorption as obtained from MD simulations (symbols) and theoretical predictions: eq 6 (solid lines), its linear expansion eq 8 (dotted line), and the second-order virial expansion eq S5 (dash-dotted lines).

S6. ADSORPTION TO SOLID SURFACES

In Figure S8, we show rescaled density profiles (as in Figure 5b in the main text), highlighting the penetration of alcohols into the surface. In Figures S9, S10, and S11, we show the comparison between MD simulations results and the Langmuir (eq 2) and Henry (eq 3) isotherms for the surfaces not shown in Figure 6. The parameters of the Langmuir isotherm, Γ_∞ and k_c, were fitted, while the coefficient K_s for Henry's law was calculated from the fitted parameters as $K_\mathrm{s} = \Gamma_\infty\, k_c$. The fitting results are shown in Figures 7 and S12.

S7. ESTIMATING SURFACTANT ADSORPTION AT THE SOLID–VAPOR INTERFACE

To check that there is no adsorption at the solid–vapor interface, we perform simulations of a cylindrical surfactant-containing water droplet on the solid surface. The surface is the same as in the main simulations but twice as long in the y-direction. The droplet is periodically replicated along the x-direction, as shown in Figure S13.

Averaging the densities over time and x-direction, we obtain the yz-resolved surfactant density profile, which is shown in Figure S14. From the obtained density plots of all three surfactants, it is possible to see that the density at the solid–vapor interface remains zero, which justifies our assumption.

References

(1) Kanduč, M. Going Beyond the Standard Line Tension: Size-Dependent Contact Angles of Water Nanodroplets. *J. Chem. Phys.* **2017**, *147*, 174701.

(2) Chodzińska, A.; Zdziennicka, A.; Jańczuk, B. Volumetric and Surface Properties of Short Chain Alcohols in Aqueous Solution–Air Systems at 293 K. *J. Solution Chem.* **2012**, *41*, 2226–2245.

(3) Basařová, P.; Váchová, T.; Bartovská, L. Atypical wetting behaviour of alcohol–water mixtures on hydrophobic surfaces. *Colloids Surf. A: Physicochem. Eng. Asp.* **2016**, *489*, 200–206.

(4) Posner, A.; Anderson, J.; Alexander, A. The surface tension and surface potential of aqueous solutions of normal aliphatic alcohols. *J. Colloid Sci.* **1952**, *7*, 623–644.

(5) Butt, H.-J.; Graf, K.; Kappl, M. *Physics and Chemistry of Interfaces*; Wiley, 2003.

(6) Paine, C.; Seidel, G. Second Virial Coefficient of Helium Adsorbed on Liquid Hydrogen. *Physi. Rev. B* **1994**, *50*, 3134.

(7) Kanduč, M.; Netz, R. R. Atomistic Simulations of Wetting Properties and Water Films on Hydrophilic Surfaces. *J. Chem. Phys.* **2017**, *146*, 164705.

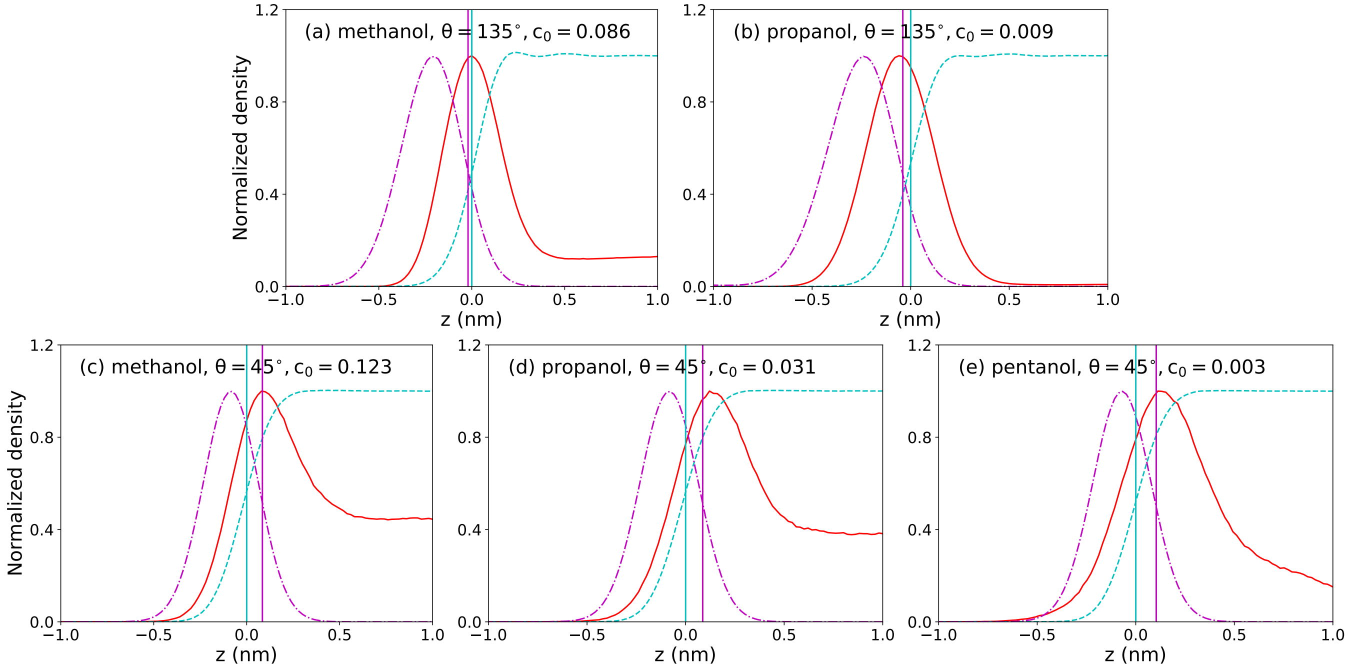

Figure S8: Rescaled density profiles of surfactants (solid red lines), the surface OH groups (magenta dash-dotted lines), and water (cyan dashed lines). Effective phase boundaries are depicted by the Gibbs dividing surface for water (cyan) and the position at half height on the water side of the OH group (magenta). The values of c_0 are in mol/l.

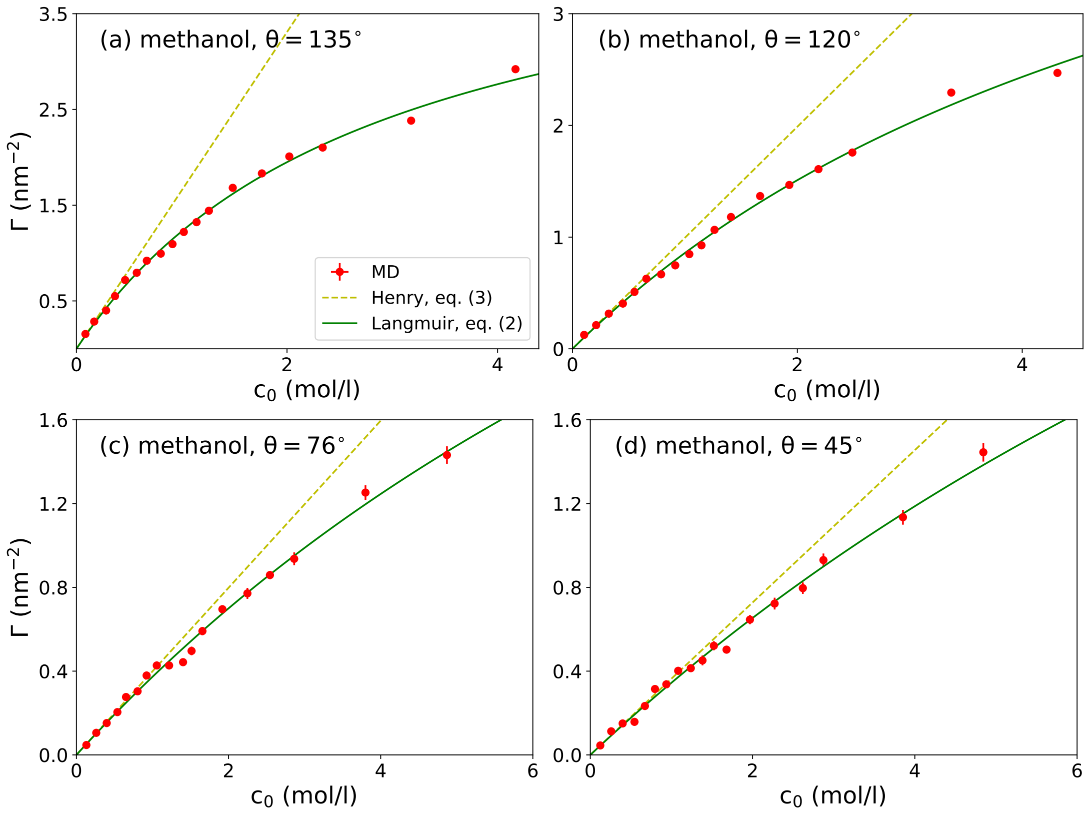

Figure S9: Adsorption Γ at the solid–water interface as a function of surfactant concentration for various water contact angles. MD values are shown by red circles, whereas solid green lines show the fits of the Langmuir isotherm. Yellow dashed lines correspond to Henry's law (eq 3), for which the coefficient K_v is taken from the Langmuir fit.

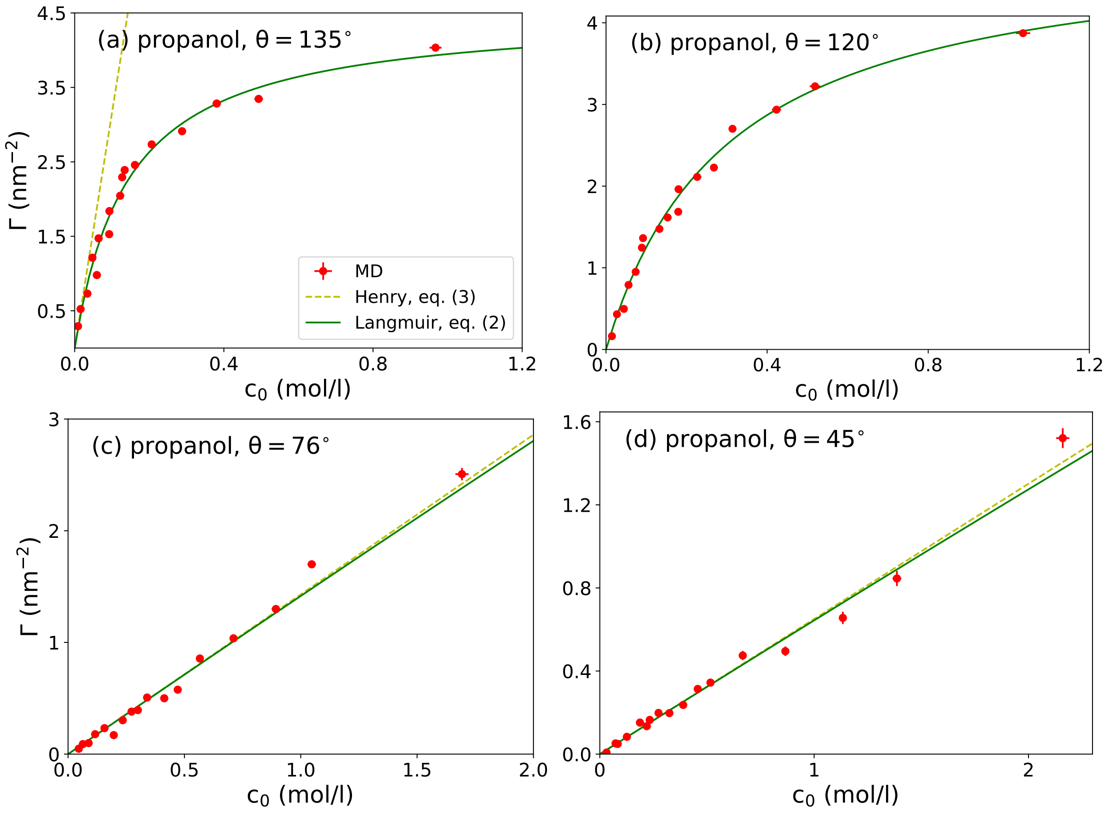

Figure S10: Same as Figure S9 but for propanol.

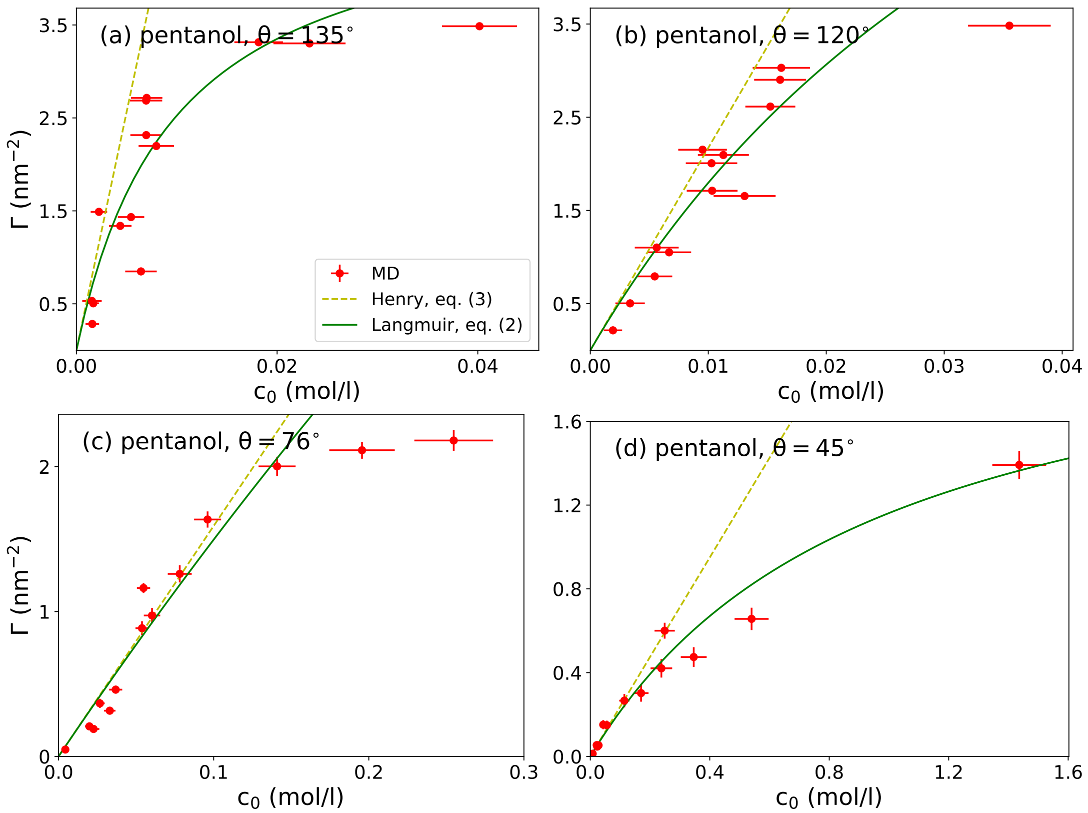

Figure S11: Same as Figure S9 but for pentanol.

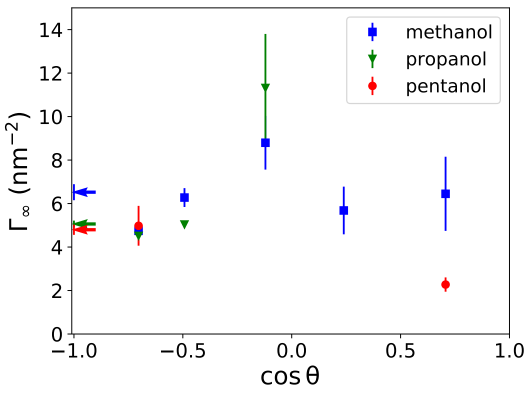

Figure S12: The fitted saturation value Γ_∞ from the Langmuir fit *versus* the surface wetting coefficient for the three alcohols. The arrows on the left represent Γ_∞ for the water–vapor interface.

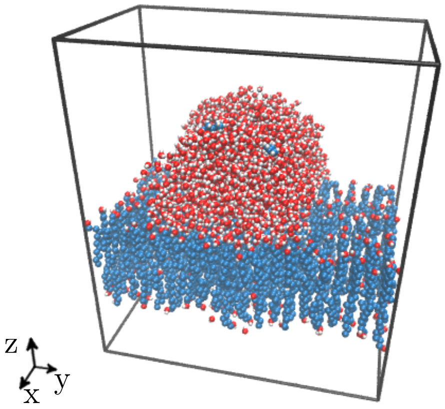

Figure S13: Simulation snapshot of a cylindrical surfactant-containing water droplet on a planar solid surface, used to estimate the amount of the surfactant at the solid–vapor interface.

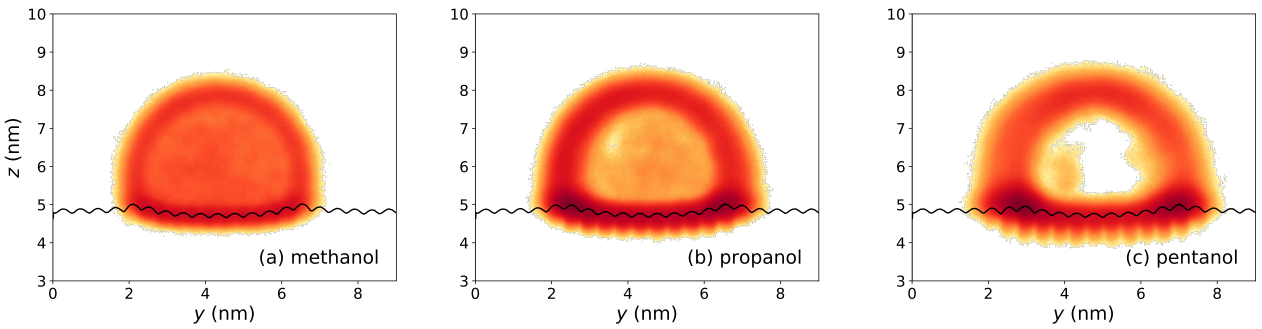

Figure S14: Side-view surfactant density plots of cylindrical water droplets for (a) methanol, (b) propanol, and (c) pentanol. The color scale runs from white (low densities) to dark red (large densities). In all three cases, the solid surface has a contact angle of $\theta = 120°$. The black line denotes the surface boundary, defined as the position where the hydroxyl (OH) density on the water side reaches half of its maximal value.
